# Effect of Bacterial-Enzymatic Synergistic Liquid Fermented Rapeseed Meal on Growth Performance, Intestinal Health, and Muscle Development of Growing Pigs

**DOI:** 10.3390/ani16071092

**Published:** 2026-04-02

**Authors:** Jingchao Liu, Ting Zhang, Yunkai Li, Jingyi Zhang, Xiaolei Zhao, Meng Li, Guoqing Cao, Bugao Li, Xiaohong Guo, Yang Yang

**Affiliations:** 1 College of Animal Science, Shanxi Agricultural University, Jinzhong 030801, Taigu, China; 18648165075@163.com (J.L.); zhangting2301@sxau.edu.cn (T.Z.); liyunkai54@163.com (Y.L.); 19735413952@163.com (J.Z.); 13935409710@163.com (X.Z.); 13994576150@163.com (M.L.); sxndcgq@sxau.edu.cn (G.C.); bugaoli@sxau.edu.cn (B.L.); 2 Shanxi Key Laboratory of Animal Genetics Resource Utilization and Breeding, Jinzhong 030801, Taigu, China

**Keywords:** fermented rapeseed meal, growing pigs, growth performance, intestinal health, skeletal muscle development, bacterial-enzymatic synergistic

## Abstract

This study investigated the synergistic effects of liquid fermentation of rapeseed meal (RSM) on feed microbiota, growth performance, and muscle development in growing pigs. RSM was fermented using four compound probiotics and eleven enzyme preparations, and microbial changes were analyzed using 16S rRNA sequencing. Seventy-two Duroc × Jingfen White pigs were randomly assigned to three groups: soybean meal (Ctrl), RSM, and fermented RSM (FRSM). FRSM showed higher trichloroacetic acid-soluble protein (TCA-sp) content and significantly lower neutral detergent fiber (NDF), acid detergent fiber (ADF), anti-nutritional factors (ANFs), and toxins (TS) (*p* < 0.01). Fermentation increased microbial diversity, with higher abundances of *Lactobacillus* and *Pediococcus*. Compared with Ctrl and RSM, the feed-to-gain ratio (F/G) decreased in the FRSM group (*p* < 0.01). FRSM also improved serum antioxidant capacity, enhanced intestinal villus height (VH)and villus height/crypt depth ratio (VH/CD), and upregulated the expression of tight junction proteins (ZO-1, occludin) and the anti-inflammatory factor IL-10 (*p* < 0.01). FRSM group also increased myofiber diameter and cross-sectional area in the *longissimus dorsi* and elevated *MyoD*, *MyoG* and *Myf5* expression (*p* < 0.01). RNA-seq revealed 2094 differentially expressed genes enriched in metabolic pathways. Overall, FRSM improved growth performance, intestinal health, and muscle development in growing pigs, which may guide the development of protein resource utilization technologies.

## Simple Summary

Rapeseed meal is a nutrient-rich feed ingredient, containing proteins, trace elements, and sulfur-containing amino acids, making it a valuable protein alternative for animal diets. However, its high levels of anti-nutritional factors and crude fiber reduce palatability and may impair the health of monogastric animals, thereby significantly limiting its practical use. To improve its nutritional value, this study fermented rapeseed meal via bacterial-enzymatic synergistic liquid fermentation, investigating whether this process reduces levels of anti-nutritional factors and increases the abundance of beneficial bacterial genera. The study also evaluated the effects of fermented rapeseed meal on growth performance, intestinal health, and muscle development in growing pigs. The results demonstrated that fermentation significantly enhanced the nutritional value of rapeseed meal and increased beneficial bacterial genera. Including fermented rapeseed meal in the pigs’ diet led to a decrease in the feed-to-gain ratio, enhanced serum antioxidant capacity, improved intestinal morphology, and upregulated the expression of anti-inflammatory factors and tight junction proteins in the jejunum of the pigs. It also facilitated *longissimus dorsi* development by promoting the expression of muscle-related genes, including MyoD, MyoG, and Myf5.

## 1. Introduction

With the shortage of protein feed resources and rising feed costs, livestock production has moved toward alternative or unconventional feed ingredients. As a result, there is an urgent need to develop new plant-based protein sources to alleviate pressure on soybean meal supplies. Rapeseed meal (RSM) is rich in protein, trace elements, and sulfur-containing amino acids, and has a balanced essential amino acid profile [1,2]. However, its application is limited by poor nutritional digestibility and high levels of anti-nutritional factors (ANFs), including glucosinolates, phytic acid, and crude fiber. These ANFs can negatively affect the taste of RSM, induce thyroid hypertrophy, and compromise the health of livestock and poultry [3,4]. Currently, biological fermentation has emerged as a potential strategy to improve the utilization of RSM.

Alongside enhancing the nutritional value of RSM, microbial fermentation can modify amino acid composition and promote the synthesis of antioxidants [5]. Probiotics such as *Lactobacillus* produce lactic acid, inhibit mold growth in fermented feed, and help protect the intestinal barrier [6]. *Bacillus subtilis*, a well-known producer of extracellular enzymes, is used as a feed additive to improve animal intestinal function [7,8]. *Bacillus coagulans* can synthesize amino acids and probiotic enzymes while regulating immune responses [9], and *Pediococcus pentosaceus* has been shown to alleviate oxidative stress and restore the intestinal microbiota [10]. However, some probiotics lack key extracellular hydrolases, such as cellulase and pectinase, which can result in incomplete removal of ANFs [11]. To address this issue, bacterial-enzymatic synergistic fermentation has been suggested to improve the quality of RSM [12]. This process facilitates the conversion of proteins into small peptides and amino acids that are more easily digested and absorbed [13]. However, the application of bacterial-enzymatic synergistic fermentation in RSM is yet to be fully developed.

During early growth, the intestinal tract of pigs is immature. Weaning stress, dietary transitions, and other factors can impair intestinal development and negatively affect growth performance [14]. Studies have demonstrated that fermented rapeseed meal (FRSM) enhances beneficial microorganisms, thereby improving blood biochemical parameters and intestinal function in pigs [15,16]. In addition, bio-fermented feed may regulate muscle development and meat quality through the gut-muscle axis [17]. Therefore, after establishing a method for bacterial-enzymatic synergistic liquid fermentation, this study evaluated feed quality and further investigated the effects of FRSM on growth performance, intestinal health, and skeletal muscle development in growing pigs.

## 2. Materials and Methods

### 2.1. Fermented Feed Preparation

RSM was mixed with water at a ratio of 1:2.5 (*w*/*v*) and inoculated with 6% (*v*/*w*) mixed bacterial culture containing *Lactobacillus plantarum* (BNCC336421, 1.0 × 10^8^ CFU/mL), *Bacillus subtilis* (BNCC109047, 4.0 × 10^9^ CFU/mL), *Bacillus coagulans* (BNCC134487, 2.0 × 10^8^ CFU/mL), and *Pediococcus pentosaceus* (BNCC135034, 1.0 × 10^8^ CFU/mL). A 4% complex enzyme preparation (Table S1) was then added and uniformly mixed. The mixture was sealed in sterile bags and fermented at 37 °C for 3 days in an electric incubator. All bacterial strains were purchased from Beijing North Carolina Biological Technology Co., Ltd. (Beijing, China), and the forage enzyme preparations were purchased from SUNSON Biotechnology Co., Ltd. (Cangzhou, China).

The experimental diets included a control diet (Ctrl) and diets containing RSM or FRSM (Table 1). The vitamin and mineral levels in all diets were formulated to meet the requirements of the National Research Council [18].

### 2.2. Chemical Analysis and Microscopic Observation of Diets

Neutral detergent fiber (NDF), acid detergent fiber (ADF), and crude fiber (CF) were measured using an ANKOM A200i Fiber Analyzer (ANKOM Technology, Macedon, NY, USA) following the method of McRoberts and Cherney [19]. Trichloroacetic acid-soluble protein (TCA-sp) was determined following the method described by Shi [20]. Physical changes in the diets before and after fermentation were examined using scanning electron microscopy (KYKY-EM3200, Beijing, China).

### 2.3. Enzyme-Linked Immunosorbent Assay

Determination of ANFs, toxins (TS), antioxidant indices, tight junction proteins, and inflammatory cytokines was carried out according to the instructions of a commercial enzyme-linked immunosorbent assay (ELISA) kit. Kits for α-conglycinin (MM-1027O1), β-conglycinin (MM-63303O1), trypsin inhibitor (TI, MM-1037PL2), phytohemagglutinin (PHA, MM-33715O2), deoxynivalenol (DON, MM-32865O1), zearalenone (ZEN, MM-32862O1), aflatoxin B_1_ (AFB_1_, MM-0925O1), T-2 toxin (TS, MM-95284O1), immunoglobulin-A (IgA, MM-0905O1), immunoglobulin-G (IgG, MM-0403O1) were purchased from Jiangsu Meimian Industrial Co., Ltd. (Yancheng, China). Serum antioxidant indices, including malondialdehyde (MDA, A003-1-2), catalase (CAT, A007-1-1), total antioxidant capacity (T-AOC, A015-2-1), superoxide dismutase (SOD, A001-3-2), and glutathione peroxidase (GSH-PX, A005-1-2), were measured using biochemical assay kits from Nanjing Jiancheng Bioengineering Institute, (Nanjing, China). ELISA kits for interleukin-4 (IL-4, YJ31752), interleukin-6 (IL-6, YJ35656), interleukin-10 (IL-10, YJ31974), and tumor necrosis factor-α (TNF-α, YJ31476) were purchased from Shanghai MeiLian Industrial Co., Ltd., (Shanghai, China). All ELISAs were conducted with four biological replicates (*n* = 4).

### 2.4. Feed Microbial Analysis

Feed samples were sent to the Gene Denovo sequencing platform (Guangzhou, China) for 16S rRNA sequencing and data analysis. Microbial DNA was extracted using the HiPure Soil DNA Kit (Guangzhou, China), followed by polymerase chain reaction (PCR) amplification. Libraries were prepared with the Illumina DNA Prep Kit (Illumina, Inc., San Diego, CA, USA), and sequenced on a Novaseq 6000 platform (Illumina, Inc., San Diego, CA, USA) using PE250 mode [21]. Raw reads were processed with DADA2 software (v1.14.1) for data quality filtering, splicing, removal of redundancy, and noise reduction. Tag sequences were classified at the phylum and genus levels, and bar charts were created using the ggplot 2 package (version 2.2.1) in R (version 3.3.3). QIIME software (version 1.9.1) was used to analyze the alpha diversity indices, and Venn diagrams were created using the vegan package in R. Principal coordinates analysis (PCoA) was applied to classify samples and assess intergroup trends, thereby excluding poorly reproducible and outlier samples. LEfSe software(version 1.0) was used to identify significant differences between groups, providing an evolutionary branching diagram. The Tax4Fun software (version 1.0) was used for functional annotation and prediction of microbial community functions, based on the Kyoto Encyclopedia of Genes and Genomes (KEGG) pathway database.

### 2.5. Feeding Trial Design and Sample Collection

Seventy-two Duroc × Jingfen growing pigs (initial body weight: 17.77 ± 2.15 kg) were randomly assigned to three groups, with four replicates per group and six pigs per replicate (three males and three females). The pigs had free access to feed and water and were fed twice daily at 08:00 and 17:00. The total duration of the trial was 30 days, during which average daily gain (ADG), average daily feed intake (ADFI), and feed-to-gain ratio (F/G) were monitored.

Blood samples were collected from the anterior vena cava using heparin sodium tubes one day prior to slaughter to ensure consistency in sampling conditions. Following the collections, the samples were immediately placed on ice for 15–20 min to allow stabilization. The samples were then centrifuged at 3500× *g* for 15 min at 4 °C to isolate the serum, which was stored at −20 °C until further analysis.

Slaughtering was carried out at the Experimental Animal Center of the College of Animal Science and Technology, Shanxi Agricultural University, in strict accordance with animal welfare guidelines. Euthanasia was performed through electrical stunning combined with exsanguination, based on the following stunning parameters: current intensity ≥ 1.3 A, voltage 200–400 V, and stunning duration 1–3 s. Euthanasia efficacy was immediately confirmed based on the absence of the corneal reflex, cessation of rhythmic respiration, and the presence of muscular flaccidity following tonic–clonic seizures. Within 15 s of stunning, carotid arteries and jugular veins were severed for exsanguination, and the animals were placed head-down for at least 30 s to ensure complete blood drainage. Death of the pigs was subsequently reconfirmed by the presence of fixed and dilated pupils, the cessation of cardiac activity, and complete apnea.

All animal experimental procedures were approved by the Animal Care and Use Committee of Shanxi Agricultural University (SXAU-EAW-2021MS. P.052801).

### 2.6. Hematoxylin-Eosin Staining

After slaughtering, the small intestine was separated from the large intestine and divided into three segments, according to the methodology of Wu et al. [22]: the duodenum was 10 cm from the pylorus, the distal ileum was 5 cm proximal to the ileocecal junction, and the jejunum was separated from the middle segment. Samples of the duodenum, jejunum, ileum, and *longissimus dorsi* were collected. Portions of these tissues were fixed in 4% paraformaldehyde solution for hematoxylin–eosin (HE) staining to perform morphological analysis. Intestinal sections were observed under a microscope at 100× magnification, while skeletal muscle sections were examined at 40× magnification. Using ImageJ software (Image-Pro Plus 6.0, Media Cybernetics, Rockville, MD, USA), the intestinal villus height (VH) and crypt depth (CD) were measured, followed by calculation of the villus height/crypt depth ratio (V/C). In addition, the muscle fiber diameter and cross-sectional area were also determined.

### 2.7. Western Blot and Quantitative Real-Time Polymerase Chain Reaction

Total protein was extracted from *longissimus dorsi* muscle samples using a total protein extraction kit (KeyGen Biotech, Nanjing, China). Equal amounts of protein were separated by sodium dodecyl sulfate-polyacrylamide gel electrophoresis (SDS-PAGE) before being transferred onto polyvinylidene fluoride (PVDF) membranes. The membranes were then blocked with 5% non-fat milk (Sangon Biotech Co., Ltd., Shanghai, China) to prevent non-specific binding and subsequently incubated overnight at 4 °C with primary antibodies. After washing, the membranes were incubated with the appropriate secondary antibodies at room temperature for 1 h. Antibodies against MyoD (A0671) and MyoG (A17427) were obtained from Abclonal and diluted at 1:500–1:1000, while GAPDH antibody (10494-1-AP) was purchased from Proteintech and diluted at 1:20,000. Western blot analysis was performed using an Odyssey infrared imaging system (LI-COR Biosciences, Lincoln, NE, USA), with an exposure time of 30 s to ensure signal linearity. Relative protein expression levels were quantified by gray value analysis and normalized to GAPDH as the internal control. All experiments were conducted with three biological replicates (*n* = 3).

Frozen *longissimus dorsi* muscle samples were first homogenized in 1 mL of Trizol reagent (TaKaRa, Beijing, China) to extract total RNA. Following this, the purified RNA was reverse-transcribed into complementary DNA (cDNA). The relative expression levels of target genes were calculated using the 2^−ΔΔCt^ method. The oligonucleotide primer sequences for the reference gene *GAPDH* and muscle development genes (*MyoD*, *MyoG*, *Myf5*) used in quantitative real-time polymerase chain reaction (qRT-PCR) are shown in Table S2.

### 2.8. Transcriptomics Sequencing Analysis

Total RNA was extracted from the collected muscle samples and reverse-transcribed into cDNA for PCR amplification. The cDNA libraries were sequenced on an Illumina Novaseq 6000 platform by Gene Denovo biotech Co. (Guangzhou, China). Differential gene expression between the two groups was analyzed using DESeq2 software (version 1.36.0). Genes with a false discovery rate (FDR) of less than 0.05 and an absolute fold change of greater than or equal to 2 were considered differentially expressed genes (DEGs). Functional annotation was achieved using Gene Ontology (GO) analysis with the GO seq R software (version 4.2.0) package, and KEGG pathway analysis was performed using KOBAS software (version 3.0). An FDR of less than 0.05 was used to define significant enrichment of DEGs in GO terms of KEGG pathways.

### 2.9. Statistical Analysis

In this study, all data were processed using SPSS Version 26.0 (IBM Corporation, Chicago, IL, USA). Prior to performing one-way ANOVA, data normality and homogeneity of variance were assessed using the Shapiro–Wilk test and Levene’s test, respectively, and all assumptions were satisfied (*p* > 0.05). One-way ANOVA was used to determine the significance of the data, followed by Duncan’s multiple range test to compare the significance of differences among group means. All results are expressed as means ± standard error of the mean (SEM). Statistical significance was considered at *p* < 0.05.

## 3. Results

### 3.1. Fermented Feed Characteristics

**Figure 1:**
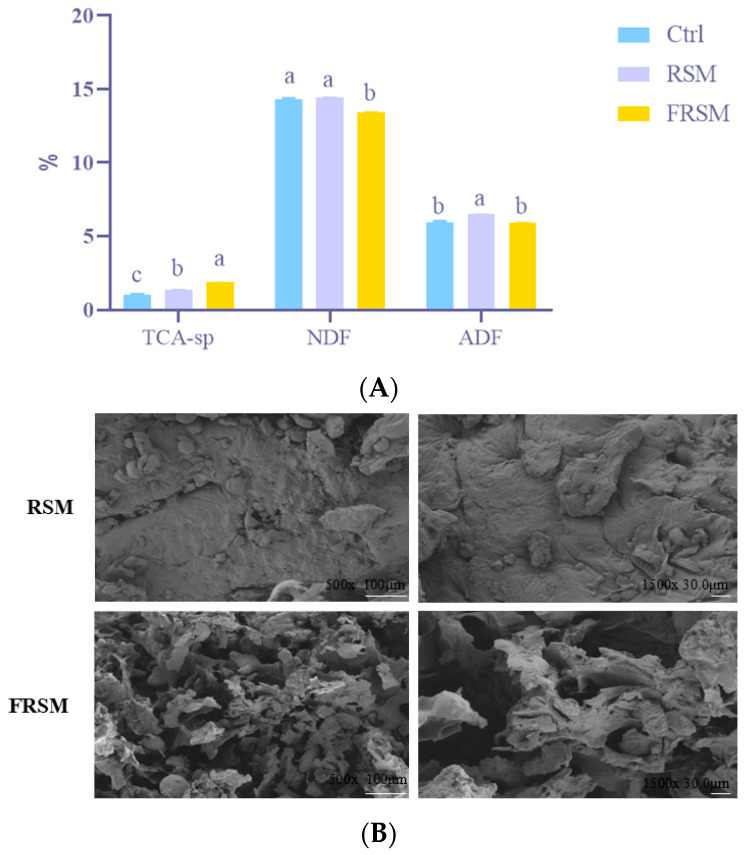
Effects of bacterial-enzymatic synergistic liquid fermentation on feed composition and surface morphology. (**A**) Nutritional components of the feed, including trichloroacetic acid-soluble protein (TCA-sp), neutral detergent fiber (NDF), and acid detergent fiber (ADF) in control (Ctrl, soybean meal), rapeseed meal (RSM), and fermented rapeseed meal (FRSM) groups. (**B**) Scanning electron microscopy images showing feed surface structure at 500× and 1500× magnification. ^a–c^ Different superscript letters indicate significant differences between mean values for a given parameter (*p* < 0.05).

RSM underwent bacterial-enzymatic synergistic fermentation to improve its nutritional profile. As shown in Figure 1A, TCA-sp was significantly higher in the FRSM group than in the Ctrl and RSM groups (*p* < 0.01). Meanwhile, the levels of NDF and ADF in the FRSM group were significantly lower than those in the RSM group (*p* < 0.01).

The levels of ANFs, including α-conglycinin, β-conglycinin, TI, and PHA, were significantly lower in the FRSM group compared with the Ctrl and RSM groups (Table 2). In addition, mycotoxins such as deoxynivalenol (DON) and zearalenone (ZEN) were significantly reduced in the FRSM group relative to both the Ctrl and RSM groups (Table 3, *p* < 0.01).

Scanning electron microscopy showed that the RSM group displayed a smooth and flat surface structure, while the FRSM group was characterized by a rough and shrunken surface with indistinct plant cell wall boundaries, increased porosity, and evidence of cell wall lysis (Figure 1B). These structural changes are likely to enhance feed digestibility and nutrient absorption in animals.

### 3.2. Feed Microbiota Characterization

**Figure 2:**
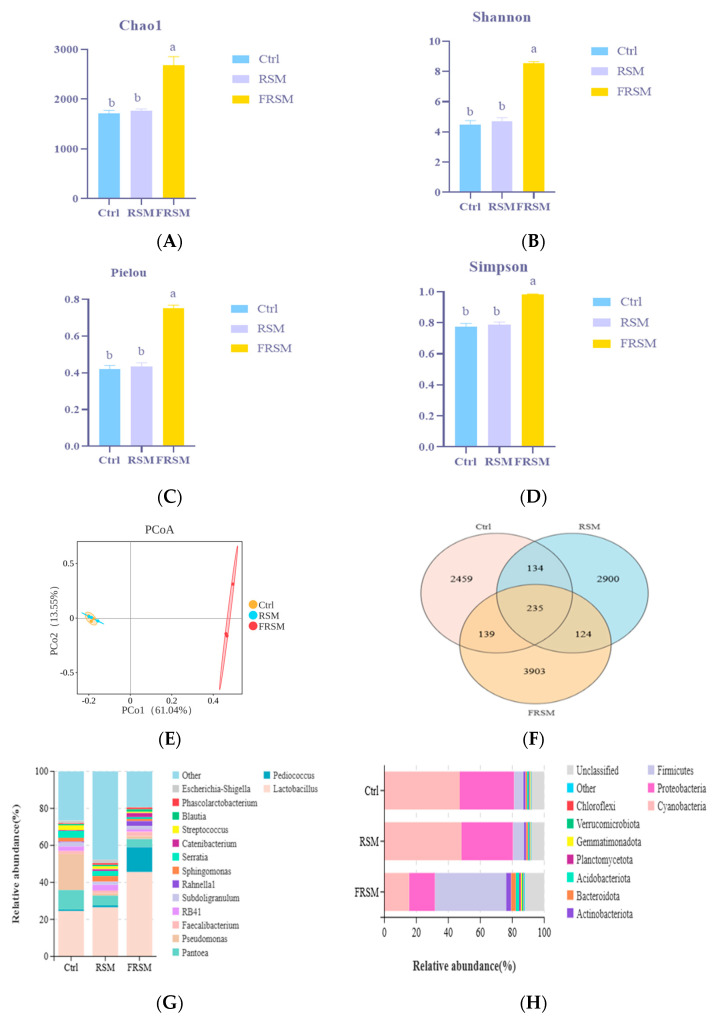

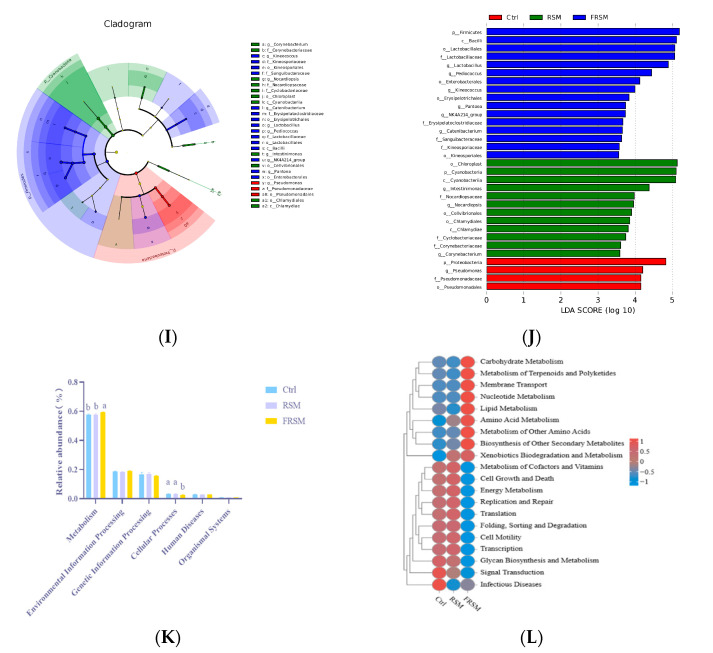
Microbial community structure and functional analysis of liquid fermented feed. Ctrl, soybean meal; RSM, rapeseed meal, FRSM, fermented rapeseed meal. (**A**–**D**) Alpha diversity indices, including Chao 1, Shannon, Pielou, and Simpson indices; (**E**) Venn diagram showing shared and unique amplicon sequence variants among groups; (**F**) Principal coordinate analysis (PCoA) of microbial community structure; (**G**) Relative abundance of bacterial phyla; (**H**) Relative abundance of bacterial genera; (**I**) Linear discriminant analysis (LDA) score distribution (LDA > 3.5); (**J**) LEfSe multilevel species hierarchical tree diagram; (**K**) Kyoto Encyclopedia of Genes and Genomes 1-level pathway analysis; (**L**) Kyoto Encyclopedia of Genes and Genomes 2-level pathway analysis. ^a,b^ Different superscript letters indicate significant differences between mean values for a given parameter (*p* < 0.05).

16S RNA sequencing analysis showed that the alpha diversity of the FRSM group was significantly higher than that in the Ctrl and RSM groups (*p* < 0.05) (Figure 2A–D). The PCoA results showed that samples within each treatment group were relatively clustered, indicating good within-group repeatability (Figure 2E). A total of 3903 unique amplicon sequence variants were identified in the FRSM group (Figure 2F). At the phylum level, the relative abundance of Firmicutes increased in the FRSM group, whereas Cyanobacteria and Proteobacteria decreased. At the genus level, the abundances of *Lactobacillus* and *Pediococcus* were significantly elevated in the FRSM group (Figure 2G,H). A total of 16 taxa showed significant differential abundance in the FRSM group, with *Lactobacillus* and *Pediococcus* identified as the main characteristic genera (Figure 2I,J). KEGG pathway analysis showed that the significantly different, metabolic pathways in the FRSM group were primarily associated with fructose and mannose metabolism, amino acid and nucleotide metabolism, starch and sucrose metabolism, and arginine and proline metabolism (Figure 2K,L).

### 3.3. Growth Performance of Pigs

Feeding with FRSM significantly reduced the F/G in the FRSM group compared with the other groups (Table 4) (*p* < 0.01).

### 3.4. Serum Biochemical Parameters, Tight Junction Proteins, and Intestinal Inflammatory Cytokines

As shown in Table 5, CAT activity was significantly higher in the FRSM group compared with the other groups (*p* < 0.01). Also, MDA levels were significantly reduced in both the RSM and FRSM groups compared to the Ctrl group (*p* < 0.05).

The concentrations of tight junction proteins, including ZO-1 and occludin, in the jejunum were significantly increased in the RSM and FRSM groups compared with the Ctrl group (*p* < 0.01). Moreover, including FRSM in the pigs’ diet significantly reduced the expression of the pro-inflammatory cytokine Il-6 (*p* < 0.05) while increasing the expression of the anti-inflammatory cytokine Il-10 (*p* < 0.01).

### 3.5. Intestinal Morphology

**Figure 3:**
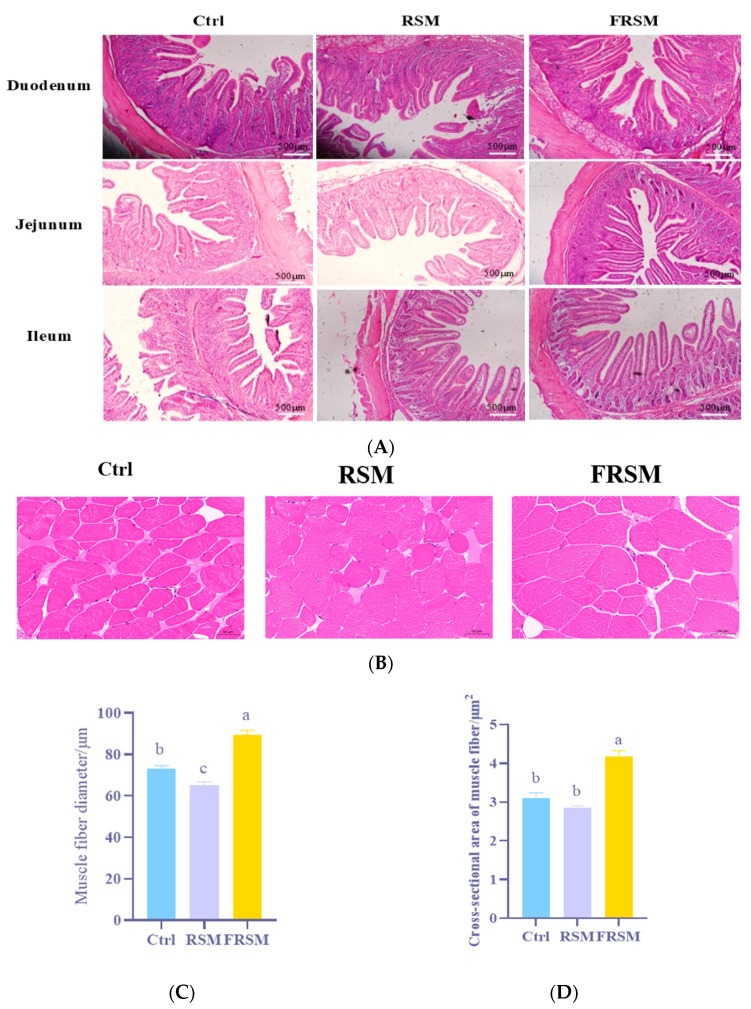

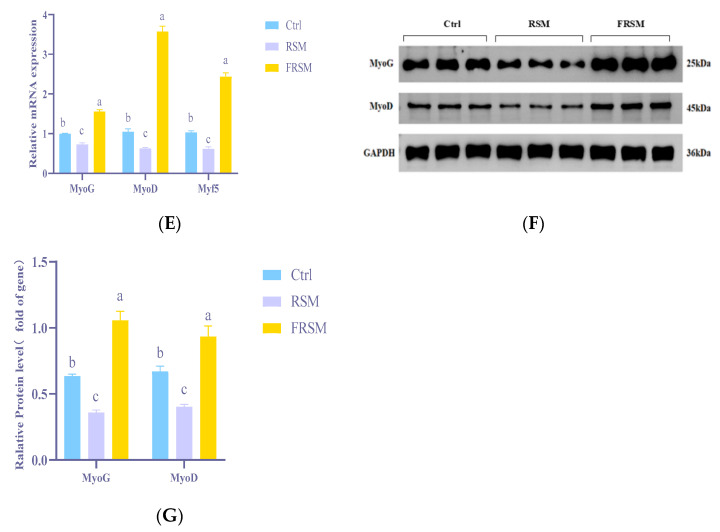
Effects of bacterial-enzymatic synergistic liquid fermented feed on intestinal morphology and skeletal muscle development in growing pigs. (**A**) Hematoxylin-eosin-stained sections of the intestine (100×); (**B**) Hematoxylin-eosin-stained sections of *longissimus dorsi* muscle (40×); (**C**) Quantitative analysis of muscle fiber diameter (μm); (**D**) Quantitative analysis of muscle fiber cross-sectional area (μm^2^); (**E**) Relative mRNA expression levels of muscle development-related genes; (**F**,**G**) Protein expression levels of muscle development-related genes (original Western blot figures are in Figure S1). Note: Ctrl, soybean meal; RSM, rapeseed meal; FRSM, fermented rapeseed meal; *MyoG*, myogenin; *MyoD*, myogenic differentiation; *Myf5*, myogenic factor 5. ^a–c^ Different superscript letters indicate significant differences between mean values for a given parameter (*p* < 0.05).

As shown in Figure 3A, dietary inclusion of FRSM significantly improved intestinal morphology, as evidenced by VH, CD, and the VH/CD. As further shown in Table 6, VH and VH/CD values in the duodenum and ileum were significantly higher in the FRSM group compared with the Ctrl and RSM groups (*p* < 0.001). In the jejunum, the FRSM group also exhibited significantly increased VH and VH/CD values (*p* < 0.001).

### 3.6. Skeletal Muscle Development

As shown in Figure 3B–D, the muscle fiber diameter and cross-sectional area in the *longissimus dorsi* of growing pigs were significantly increased in the FRSM group compared with the Ctrl and RSM groups (*p* < 0.01).

Also, including FRSM in the pigs’ diet led to a significant upregulation in the expression levels of *MyoD*, *MyoG* and *Myf5*, compared with the Ctrl and RSM groups (*p* < 0.01) (Figure 3E–G).

### 3.7. Muscle Transcriptome of Growing Pigs

**Figure 4:**
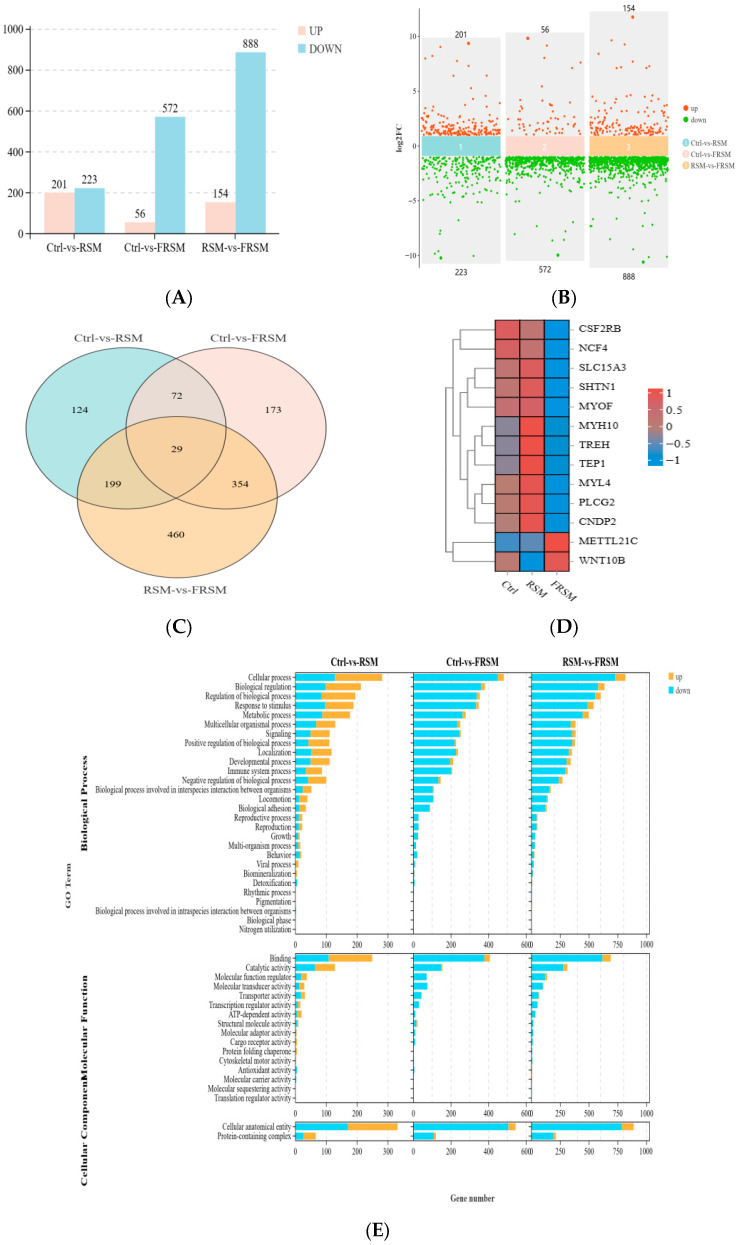

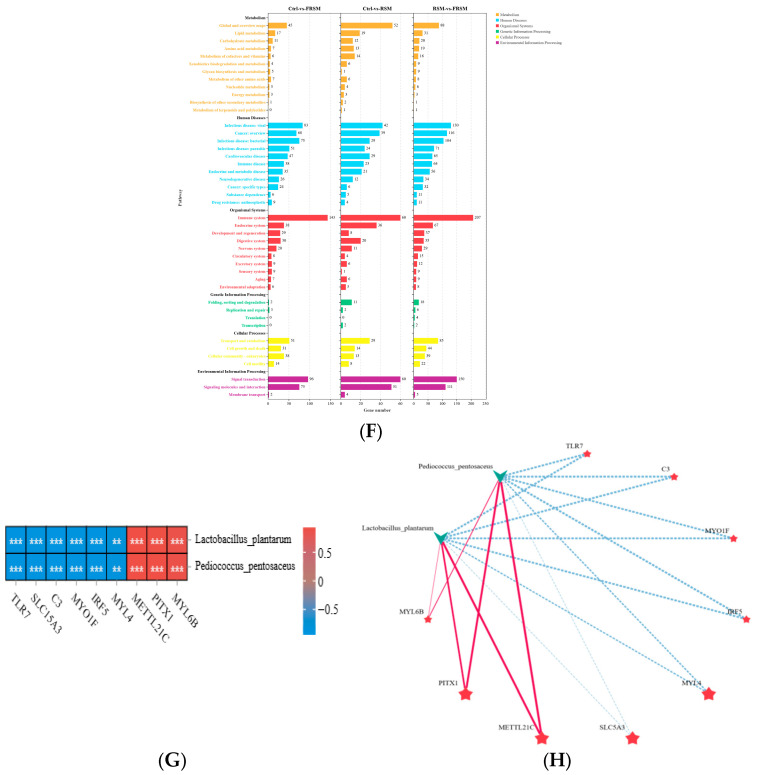
Effects of bacterial-enzymatic synergistic liquid fermented feed on the muscle transcriptome of growing pigs. Ctrl, soybean meal; RSM, rapeseed meal; FRSM, fermented rapeseed meal. (**A**) Histogram of differentially expressed genes; (**B**) Scatter plot of differentially expressed genes; (**C**) Venn diagram showing shared and unique differentially expressed genes among groups; (**D**) Cluster heatmap of differentially expressed genes; (**E**) Gene Ontology functional annotation; (**F**) Kyoto Encyclopedia of Genes and Genomes pathway enrichment analysis of differentially expressed genes; (**G**) Correlation heatmap between microbial taxa and differentially expressed genes; (**H**) Correlation network diagram. Red lines indicate positive correlations, and blue dotted lines indicate negative correlations. ** indicates *p* < 0.01 and *** indicates *p* < 0.001.

A total of 628 DEGs were identified between the Ctrl and FRSM groups, including 56 upregulated genes and 572 downregulated genes. In the comparison between the RSM and FRSM groups, 1042 DEGs were identified, of which 154 genes were upregulated and 888 genes were downregulated (Figure 4A,B). Venn diagram analysis revealed that there were 29 common DEGs shared among the three groups (Figure 4C,D). GO enrichment analysis showed that these DEGs were mainly associated with biological regulation, catalytic activity, and protein complex-related processes (Figure 4E). KEGG pathway analysis further showed that the DEGs were mainly enriched in pathways such as lipid metabolism, carbohydrate metabolism, amino acid metabolism, and energy metabolism (Figure 4F).

Pearson’s correlation analysis was performed to explore the correlation between differential microbial communities and differential genes. Among the differential bacterial species, *Lactobacillus plantarum* and *Pediococcus pentosaceus* were selected for correlation analysis with DEGs. The results showed that *Lactobacillus plantarum* and *Pediococcus pentosaceus* were significantly positively correlated with *METT21C*, *PITX1* and *MYL6B* (*p* < 0.01), whilst being negatively correlated with *MYL4*, *SLC15A3*, *IRF5*, *C3*, *TLR7*, and *MYO1F* (*p* < 0.01) (Figure 4G,H).

## 4. Discussion

Rapeseed has long been regarded as an ideal source of animal protein due to its high protein content and balanced profile of essential amino acids. However, its application in animal nutrition is limited by the presence of a variety of ANFs [23], as well as relatively lower digestibility and utilization efficiency compared with soybean meal [24]. Fermentation has emerged as an effective strategy to combat these limitations, as it involves microbes breaking down anti-nutritional substances, inducing the synthesis of various bioactive compounds, particularly antioxidants, and facilitating the decomposition of structural components such as cellulose, pectin, and lignin [25]. Therefore, improving the feeding value of RSM has attracted considerable research interest on a global scale.

In this study, rapeseed was pretreated using bacterial-enzymatic synergistic liquid fermentation with compound probiotics and enzyme complexes. As shown in previous studies, fermentation of rapeseed significantly improved the growth of pigs, but the mechanism of action remains unclear [26]. The current findings highlight an increase in the nutrient content of FRSM, as evidenced by increased TCA-SP and reduced levels of NDF, ADF, ANFs, and TS. Additionally, the resulting fragmented surface structure of the feed is more conducive to nutrient absorption. These improvements in both nutritional composition and physical structure contributed to enhanced digestibility and animal growth performance. Furthermore, fermentation increased the abundance of beneficial microorganisms, which may further promote nutrient digestibility and growth performance. Some studies have shown that Firmicutes have a strong ability to depolymerize dietary fiber and promote digestion and absorption [27]. In other findings, feeding pigs with feed co-fermented by *Lactobacillus* and *Pediococcus* has been reported to improve feed conversion ratio [28]. Therefore, the noted decrease in F/G in growing pigs in the present study is likely associated with the increase in both nutrient availability and beneficial microbial populations in the fermented feed.

A growing number of studies have confirmed that an increase in beneficial microorganisms in feed is an effective strategy for improving intestinal health [29]. *Lactobacillus* species contribute to the regulation of intestinal homeostasis by exerting antioxidant activity and modulating immune function [30], while *Pediococcus* has also been shown to possess positive effects on intestinal health [31]. As the primary site for nutrient digestion and absorption in animals, the intestine plays a key role in determining nutrient utilization efficiency and overall growth performance. Moreover, the intestinal mucosal layer serves as the first line of defense against pathogenic microbial colonization, and its integrity is integral for maintaining intestinal homeostasis. Tight junction proteins, such as ZO-1 and occludin, are key components of the intestinal barrier, and the digestive and absorptive capacity of the intestine is largely dependent on its morphological structure [32].

In the present study, VH and the VH/CD in the jejunum, ileum, and duodenum were significantly increased in growing pigs fed FRSM. Additionally, the concentrations of the tight junction proteins (ZO-1 and occludin) in the jejunum were significantly elevated, indicating improved intestinal barrier integrity. In terms of intestinal immunity, the level of the pro-inflammatory cytokine IL-6 in the jejunum decreased, while the anti-inflammatory cytokine IL-10 increased. These findings are consistent with those of Jiang et al., whereby it was demonstrated that fermented feed can alleviate intestinal inflammation in growing pigs by modulating small intestinal inflammatory factor IL-6 [33]. Taken together, these results indicate that FRSM can reduce inflammatory damage in the intestines by regulating intestinal immunity. Therefore, by increasing the abundance of beneficial microorganisms in feed, intestinal health is improved through optimization of intestinal morphology, enhancement of barrier function, and regulation of immune homeostasis. In turn, these effects promote more efficient nutrient digestion and absorption in growing pigs.

Existing studies have shown that improving intestinal barrier function can enhance nutrient utilization efficiency and reduce inflammatory responses, thereby promoting skeletal muscle development [34]. For instance, intestinal inflammation can induce muscle atrophy by increasing pro-inflammatory cytokines such as IL-6 and TNF-α [35], while improvements in intestinal barrier integrity and gut microbial balance can support muscle metabolism by increasing nutrient absorption efficiency and producing beneficial microorganisms. In the present study, it was found that while FRSM improved intestinal health, it also significantly upregulated the expression of myogenic marker genes, including *MyoD*, *MyoG*, and *Myf5*, along with their corresponding protein levels in muscle tissue. These findings indicate that the promotive effects of FRSM on muscle development may be somewhat related to improvements in intestinal function, which could serve as a key upstream driver in this process. Previous research has confirmed that skeletal muscle growth and functional maintenance can be effectively regulated through amino acid metabolism [36]. This is consistent with the KEGG enrichment results found in this study, which further verifies the crucial role of amino acid metabolism in muscle function regulation. In addition, correlation analysis found that *METT21C*, *PITX1*, and *MYL6B* were significantly positively correlated with *Lactobacillus plantarum* and *Pediococcus pentosaceus*, while *MYL4*, *SLC15A3*, *IRF5*, *C3*, *TLR7*, and *MYO1F* showed a significant negative correlation with these two bacterial species. Existing studies have implicated many of these genes in muscle development. For instance, methyltransferase-like protein 21C (*METT21C*) has been shown to regulate protein synthesis and myoblast proliferation by mediating the modification of IGF2BP1 through lysine trimethylation [37]. Elsewhere, it was found that the direct myogenic targets of *PITX1* can activate myogenic determination genes such as *MyoD*, thereby promoting myogenesis [38]. Among the negatively correlated genes, *SLC15A3* expression has been shown to decrease in mouse tibialis anterior muscle after oral administration of carnosine [39], while *MYL4* can inhibit the proliferation of C2C12 cells, thereby influencing muscle development [40].

The complex enzyme preparations used in this study are conventional industrial feed-grade enzymes with relatively low procurement costs. Notably, dietary supplementation with FRSM significantly reduced the F/G. Considering the reduction in feed costs and the improvement in feed conversion efficiency, the overall economic benefits are likely to be comparable or even superior to those of conventional diets, demonstrating the economic feasibility and practical application potential of this approach for commercial production.

Overall, the results of this study highlight that feeding with FRSM improves growth performance, intestinal health, and muscle development in growing pigs.

## 5. Conclusions

RSM subjected to bacterial-enzymatic synergistic liquid fermented using a combination of *Bacillus subtilis*, *Pediococcus pentosaceus*, *Lactobacillus plantarum*, *Bacillus coagulans*, and an enzyme complex presented improved nutrient availability. This was reflected in the enhancement of nutritional composition, growth performance, and muscle development, as well as strengthened intestinal barrier integrity and function in growing pigs. These findings suggest that FRSM represents a potential alternative protein source for animal nutrition and offers an effective strategy for improving feed efficiency in the livestock industry.

## Figures and Tables

**Table 1:** Composition and nutrient content of experimental diets.

Ingredient	Ctrl (%)	RSM (%)	FRSM (%)
Corn	67.45	64.72	64.72
Whey powder	3.00	3.00	3.00
Soybean oil	1.23	2.22	2.22
Soybean	20.61	12.45	12.45
Fish meal	2.00	2.00	2.00
Lysine hydrochloride	0.55	0.63	0.63
Met	0.23	0.19	0.19
Thr	0.23	0.24	0.24
Trp	0.08	0.09	0.09
Limestone	0.48	0.41	0.41
CaHPO_4_	1.57	1.50	1.50
NaCl	0.56	0.55	0.55
Premix ^1^	2.00	2.00	2.00
Rapeseed meal	-	10.00	-
Fermented Rapeseed meal	-	-	10.00
Total	100.00	100.00	100.00
DM	87.19	87.34	87.34
Water	12.81	12.66	12.66
CP	17.00	16.85	16.85
EE	4.10	3.36	3.36
CF	1.92	2.73	2.73
Ash	1.14	1.13	1.13
Ca	0.69	0.69	0.69
P	0.65	0.69	0.69
SIDLys	1.20	1.20	1.20
SIDM + C	0.71	0.71	0.71
SIDThr	0.76	0.76	0.76
SIDTrp	0.24	0.24	0.24

^1^ In the premix, each kilogram of feed provides: Vitamin A 7000 IU, Vitamin D 2250 IU, Vitamin E 60 IU, Vitamin C 1.8 mg, Vitamin K3 10 mg, Vitamin B1 6 mg, Vitamin B2 18 mg, Vitamin B6 9 mg, Biotin 0.24 mg, Pantothenic acid 17 mg, Niacin 30 mg, Copper (Copper sulfate pentahydrate) 25 mg, Manganese (Manganese sulfate) 15 mg, Iron (Ferrous sulfate) 80 mg, Iodine (Potassium iodide) 0.45 mg, Selenium (Sodium selenite) 0.3 mg. Abbreviations: Ctrl, soybean meal; RSM, rapeseed meal; FRSM, fermented rapeseed meal; Met, methionine; Thr, threonine; Trp, tryptophan; CaHPO_4_, calcium hydrogen phosphate; NaCl, sodium chloride; DM, dry matter; CP, crude protein; EE, ether extract; CF, crude fiber; Ca, calcium; P, phosphorous; SIDLys, standardized ileal digestible lysine; SIDM + C, standardized ileal digestible methionine + cysteine; SIDThr, standardized ileal digestible threonine; SIDTrp, standardized ileal digestible tryptophan.

**Table 2:** Effects of bacterial-enzymatic synergistic liquid fermentation on anti-nutritional factors in feed.

Items	Ctrl	RSM	FRSM	SEM	*p*-Value
a-conglycinin (µg/g)	25.09 ^a^	21.89 ^b^	14.34 ^c^	1.133	<0.001
β-conglycinin (µg/g)	48.79 ^a^	49.54 ^a^	30.87 ^b^	2.751	<0.001
TI (µmol/L)	69.87 ^a^	56.12 ^b^	48.11 ^c^	2.601	<0.001
PHA (ng/L)	588.87 ^a^	533.14 ^b^	249.66 ^c^	36.529	<0.001

Note: SEM, standard error of the mean; Ctrl, soybean meal; RSM, rapeseed meal; FRSM, fermented rapeseed meal; TI, trypsin inhibitor; PHA, phytohemagglutinin. ^a–c^ Different superscript letters indicate significant differences between mean values for a given parameter (*p* < 0.05).

**Table 3:** Effects of bacterial-enzymatic synergistic liquid fermentation on toxin levels in feed.

Toxin (μg/kg)	Safety Standards (μg/kg)	Ctrl	RSM	FRSM	SEM	*p*-Value
DON	<1000	375.67 ^a^	295.00 ^b^	248.33 ^c^	19.449	<0.001
ZEN	<500	140.33	51.67	<20		
AFB1	<10	1.6	1.6	<1.5		
TS	<500	< 50	<50	<50		

Note: SEM, standard error of the mean; Ctrl, soybean meal; RSM, rapeseed meal; FRSM, fermented rapeseed meal; DON, deoxynivalenol; ZEN, zearalenone; AFB_1_, aflatoxin B_1_; TS, T-2 toxin. ^a–c^ Different superscript letters indicate significant differences between mean values for a given parameter (*p* < 0.05).

**Table 4:** Effects of bacterial-enzymatic synergistic liquid fermented feed on growth performance in growing pigs.

Item	Ctrl	RSM	FRSM	SEM	*p*-Value
IBW (kg)	17.48	17.92	17.93	0.16	0.485
FBW (kg)	35.31	35.81	37.58	0.57	0.250
ADG (kg/d)	0.60	0.60	0.66	0.015	0.152
ADFI (kg/d)	1.08	1.17	1.03	0.02	0.305
F/G	1.82 ^b^	1.96 ^a^	1.73 ^c^	0.03	0.002

Note: IBW, initial body weight; FBW, final body weight; ADG, average daily gain; ADFI, average daily feed intake; F/G, feed to gain ratio. ^a–c^ Different superscript letters represent significant differences among groups (*p* < 0.05).

**Table 5:** Effects of bacterial-enzymatic synergistic liquid fermented feed on serum antioxidant indices, inflammatory cytokines, and jejunal tight junction proteins in growing pigs.

Item	Ctrl	RSM	FRSM	SEM	*p*-Value
serum					
IL-10 (ng/L)	225.80	229.42	212.73	4.083	0.223
IL-6 (ng/L)	1283.88	1290.50	1274.79	13.676	0.906
IL-4 (ng/L)	102.14	99.89	102.36	1.282	0.711
CAT (U/mL)	15.59 ^b^	10.85 ^b^	23.73 ^a^	2.001	0.004
SOD (U/mL)	21.66	22.94	18.77	2.306	0.801
T-Aoc (umol Trolox/mL)	0.94	0.77	0.78	0.068	0.559
GSH-PX (nmol/min/mL)	4.11	3.75	5.72	0.675	0.511
MDA (nmol/L)	2.19 ^a^	1.06 ^b^	0.58 ^b^	0.32	0.024
jejunum					
Occludin (pg/mL)	448.41 ^b^	537.92 ^a^	553.08 ^a^	17.82	0.003
ZO-1 (pg/mL)	193.57 ^b^	237.43 ^a^	224.32 ^a^	6.44	0.005
IL-4 (ng/L)	106.40	101.11	117.46	3.25	0.101
IL-6 (ng/L)	1108.65 ^b^	1465.36 ^a^	1111.20 ^b^	71.31	0.043
IL-10 (ng/L)	275.59 ^b^	322.08 ^a^	353.91 ^a^	11.91	0.008

Note: SEM, standard error of mean; Ctrl, soybean meal; RSM, rapeseed meal; FRSM, fermented rapeseed meal; IL-4, interleukin-4; IL-6, interleukin-6; IL-10, interleukin-10; CAT, catalase; SOD, superoxide dismutase; T-Aoc, total antioxidant capacity; GSH-PX, glutathione peroxidase, ZO-1, zonula occludens-1. ^a,b^ Different superscript letters indicate significant differences between mean values for a given parameter (*p* < 0.05).

**Table 6:** Effects of fermented rapeseed meal on small intestinal morphology in growing pigs.

Item		Ctrl	RSM	FRSM	SEM	*p*-Value
Duodenum	VH (μm)	458.41 ^b^	450.82 ^b^	534.53 ^a^	8.38	<0.001
	CD (μm)	427.69	459.63	413.69	8.38	0.078
	VH/CD	1.07 ^b^	0.98 ^b^	1.29 ^a^	0.04	<0.001
Jejunum	VH (μm)	437.82 ^a^	337.11 ^b^	443.65 ^a^	0.05	<0.001
	CD (μm)	421.69 ^a^	314.26 ^b^	318.90 ^b^	0.08	<0.001
	VH/CD	1.04 ^b^	1.07 ^b^	1.39 ^a^	0.15	<0.001
Ileum	VH (μm)	384.73 ^b^	400.94 ^b^	495.82 ^a^	7.63	<0.001
	CD (μm)	323.02 ^b^	377.37 ^a^	280.32 ^c^	7.40	<0.001
	VH/CD	1.31 ^b^	1.15 ^b^	1.91 ^a^	0.05	<0.001

Note: SEM, standard error of mean; Ctrl, soybean meal; RSM, rapeseed meal; FRSM, fermented rapeseed meal; VH, villus height; CD, crypt depth; VH/CD, villus height-to-crypt depth ratio. ^a–c^ Different superscript letters indicate significant differences between mean values for a given parameter (*p* < 0.05).

## Data Availability

The raw 16S rRNA gene sequencing data generated in this study have been deposited in the NCBI SRA database under BioProject accession number PRJNA1333670. The raw transcriptome sequencing data are also available in the NCBI SRA database under BioProject accession number PRJNA1335602.

## Supplementary Materials

The following supporting information can be downloaded at: https://www.mdpi.com/article/10.3390/ani16071092/s1, Table S1: Characteristics of the forage enzyme preparation; Table S2: Primer sequences of qPCR. Figure S1. Original figures of Western blot.
