# Supplementary material for: Effect of Bacterial-Enzymatic Synergistic Liquid Fermented Rapeseed Meal on Growth Performance, Intestinal Health, and Muscle Development of Growing Pigs
Source: Animals (Basel). 2026 Apr 2;16(7):1092. doi: 10.3390/ani16071092 (PMC13072280; doi:10.3390/ani16071092)
Supplement: Supplementary file 1 [file animals-16-01092-s001.zip › animals-4167763-supplementary.pdf]

*Supplementary Material*

**1 Supplementary Tables and Figure**

**Table S1.** Characteristics of forage enzyme preparation

| Enzyme | Addition amount（g/kg） | Temperature range（℃） | Enzyme activity（U/g） |
| --- | --- | --- | --- |
| β-glucanase | 0.020 | 30-40 | ≥50000 |
| Alkaline protease | 0.010 | 40-50 | ≥200000 |
| Acidic protease (H type) | 0.025 | 30-40 | ≥60000 |
| Neutral protease | 0.015 | 30-40 | 60000 |
| $\alpha$-amylase | 0.030 | 30-40 | ≥3000 |
| Pectinase | 0.001 | 30-40 | 30000 |
| Lipase | 0.200 | 40-55 | ≥10000 |
| $\alpha$-galactosidase | 0.030 | 30-40 | ≥500 |
| Cellulase (H-type) | 0.010 | 30-40 | ≥10000 |
| Xylanase | 0.003 | 40-55 | ≥1000000 |
| Glucose oxidase | 0.005 | 30-40 | ≥10000 |

**Table S2.** Primer sequences of qPCR

| Genes | Primer sequences (5' - 3') | Product length (bp) |
| --- | --- | --- |
| *MyoD* | F:CCGTGTTTCGACTCACCAGA<br>R: GTAGTAGGCGGTGTCGTAGC | 594 |
| *MyoG* | F:GAGGAAGTCTGTGTCGGTGG<br>R:CCACGATGGACGTAAGGGAG | 395 |
| *Myf5* | F:GAGCCAGACCTCAGAAAGGAAG<br>R:CGGTGGCCTTCTGGATAAACT | 407 |
| *GAPDH* | F：GCCTCCTCCAATTCAACCCT<br>R：CTCGTGGTTCACACCCATCA | 514 |

*MyoG*, Myogenin; GAPDH, Glyceraldehyde-3-phosphate dehydrogenase; *MyoD*, Myogenic Differentiation.

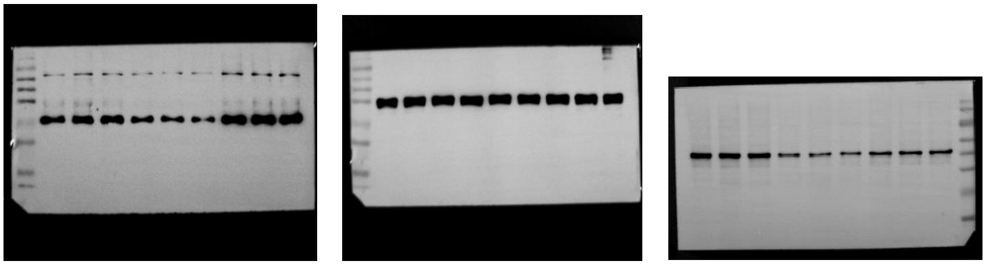

**Figure S1.** Original figures of Western blot.

All complete scans of the full original gels shown in the manuscript, together with the original files of any microscopy images included in the figures, have been submitted separately in ZIP file format to the Supplementary Material.
